# The value of a novel three-dimensional mitral valve index in the assessment of the haemodynamic severity of rheumatic mitral stenosis

**DOI:** 10.1186/s44156-025-00094-z

**Published:** 2025-11-12

**Authors:** Rodrigo Tobias Giffoni, Judy Hung, João da Rocha Medrado Neto, Airandes de Sousa Pinto, Nayana F. A. Gomes, Alexandre Negrão Pantaleão, William Antonio de Magalhães Esteves, Jacob P. Dal-Bianco, Timothy C. Tan, Robert Levine, Maria Carmo Pereira Nunes

**Affiliations:** 1 Postgraduate Course of Infectious Diseases and Tropical Medicine, School of Medicine, Federal University of Minas Gerais, Belo Horizonte, MG Brazil; 2 Massachusetts General Hospital, Harvard Medical School, Boston, MA USA; 3 State University of Feira de Santana, Feira de Santana, BA Brazil; 4 Hospital das Clínicas, Federal University of Minas Gerais, Belo Horizonte, Minas Gerais Brazil; 5 Department of Cardiology, Blacktown Hospital, University of Western Sydney, Sydney, NSW Australia; 6 Department of Internal Medicine, School of Medicine of the Federal, University of Minas Gerais, Av. Professor Alfredo Balena, 190, Santa Efigênia, Belo Horizonte, 30130-100 MG Brazil

**Keywords:** Rheumatic heart disease, Mitral stenosis, Mitral valve disease, Mitral geometry, 3-dimensional echocardiography, Valvular obstruction

## Abstract

**Background:**

Rheumatic mitral stenosis (MS) is characterised by structural alterations that reduce the size of the valvular orifice. In addition, changes in valve geometry may have haemodynamic consequences that extend beyond the narrowed orifice, influencing the overall clinical presentation of MS. The aim of this study was to develop an index to assess the haemodynamic severity of the stenosis.

**Methods:**

A total of 186 patients with rheumatic MS who underwent comprehensive three-dimensional (3D) transoesophageal echocardiographic assessment were included. Dedicated software was used to extract a range of morphological variables to evaluate mitral valve geometry, including diameter, area, height, volume, and the aortic–mitral angle. To quantify the volume enclosed within the stenotic structure, we developed the 3D Doming Index (DI), calculated by dividing the valvular volume (tenting volume) by the theoretical volume of a cylinder generated by the mitral annulus and valvular height (tenting height). Linear regression models were employed to identify determinants of the mean pressure gradient.

**Results:**

The 3D Doming Index demonstrated a significant association with the transmitral pressure gradient in the multivariate model, after adjusting for confounders including age, sex, heart rate, pulmonary artery systolic pressure, net atrioventricular compliance (C_n_), and left atrial volume. Incorporation of the 3D Doming Index into the model improved overall performance.

**Conclusions:**

The geometric configuration of the mitral valve contributes to the haemodynamic burden of obstruction in rheumatic MS. The 3D Doming Index offers valuable insight into the relationship between valve anatomy and the resultant haemodynamic impact of the stenosis.

**Supplementary Information:**

The online version contains supplementary material available at 10.1186/s44156-025-00094-z.

## Introduction

Mitral stenosis (MS) is a prevalent form of valvular heart disease globally and is primarily caused by rheumatic heart disease, which remains the most common underlying aetiology worldwide [1]. The inflammatory process associated with rheumatic fever leads to fibrosis and scarring of the mitral leaflets, resulting in structural distortion, valvular obstruction, and marked anatomical changes [2, 3]. Typically, the mitral valve adopts a domed configuration, with the anterior leaflet exhibiting the characteristic “hockey stick” appearance [4]. Consequently, the valve orifice narrows, and the mitral apparatus transitions from a tubular conduit into a funnel-shaped structure [5].

The mitral valve is a complex and dynamic structure [6]. Its annulus is normally saddle-shaped, with the commissures located at the lowest points. The annular dimensions vary throughout the cardiac cycle, decreasing during ventricular systole [7]. As a result, the mitral orifice is not strictly planar, and irregularities may be observed along the leaflet edges, including thickening, asymmetry, and distortion, features frequently observed in rheumatic heart disease.

The advent of three-dimensional (3D) echocardiography has significantly advanced our understanding of mitral valve anatomy, function, and pathology [6, 7]. In a previous study, Mahmoud Elsayed et al. proposed a technique for measuring mitral valve area using 3D transoesophageal echocardiographic imaging, which incorporates the commissures in the assessment [8]. This method enabled not only more accurate evaluation of the valve area but also reconstruction of its native saddle-shaped geometry [9]. In a separate investigation, Gilon et al. used 3D echocardiography and stereolithography to examine how mitral valve geometry affects pressure and flow dynamics [10]. Their study modelled three geometric configurations, domed, intermediate, and flat, and demonstrated that changes in valve shape could increase transmitral pressure gradients by up to 40%, particularly in valves with a flattened profile.

While previous research has addressed various aspects of mitral valve anatomy and function, the specific relationship between the three-dimensional geometry of rheumatic MS and the resulting pressure gradients has not been fully explored. This study was therefore designed to address this knowledge gap by developing a novel index to assess the haemodynamic severity of MS.

## Methods

### Study population

This was a prospective study involving patients with rheumatic MS who underwent clinically indicated three-dimensional transoesophageal echocardiography (3D-TEE) at our institution. Patients with significant concomitant mitral regurgitation or aortic valve disease, which could interfere with accurate assessment of the mitral valvular apparatus, were excluded. In addition, 13 patients were excluded due to technical limitations in the 3D-TEE images that prevented adequate visualisation of the complete mitral and aortic annuli.

Clinical data was collected using a standardised form and included sociodemographic characteristics, New York Heart Association (NYHA) functional class, and medication history. At the time of 3D-TEE acquisition, cardiac rhythm and heart rate were recorded, and the presence of atrial fibrillation was confirmed via a 12-lead electrocardiogram. The study protocol was approved by the institutional review board of the Federal University of Minas Gerais, Brazil. Written informed consent was obtained from all participants.

### Image acquisition and analysis

A comprehensive transthoracic 2D echocardiogram was performed using commercially available equipment, in accordance with established guidelines [11]. The morphological features of the mitral valve (MV) were assessed and categorised using both the Wilkins score [12] and the revised echocardiographic score, as previously described [13]. Net atrioventricular compliance (C_n_) was calculated using the following formula: determined by the formula: C_n_ (mL/mm Hg) = 1270× (planimetric MV area [cm^2^]/E-wave downslope [cm/s^2^]) [14]. In patients with non-linear diastolic flow profiles, the mid-diastolic portion of the E-wave was used, as it more accurately reflects the severity of stenosis rather than early left atrial depressurisation. When necessary, the slope was extrapolated back to estimate the initial peak velocity.

For patients in sinus rhythm, each echocardiographic parameter was averaged over three consecutive cardiac cycles. In cases of atrial fibrillation, measurements were averaged over five cycles to ensure reliability.

All patients underwent 3D-TEE under mild sedation using an iE33 or EPIQ 7 ultrasound system and an X7-2t transducer (Philips Medical Systems, Andover, MA). During image acquisition, the transoesophageal probe was positioned at the mid-oesophageal level to ensure complete visualisation of the mitral valve. To optimise image quality and volume rate, the mid- and apical segments of the left ventricle were excluded from the field of view.

A multibeat acquisition protocol (typically 2 to 6 cardiac cycles) was employed to maximise volume rate and spatial resolution. For patients with atrial fibrillation or those unable to hold their breath, a single-beat real-time 3D zoomed mode was used to minimise stitching artefacts and enhance image clarity.

**Fig. 1:**
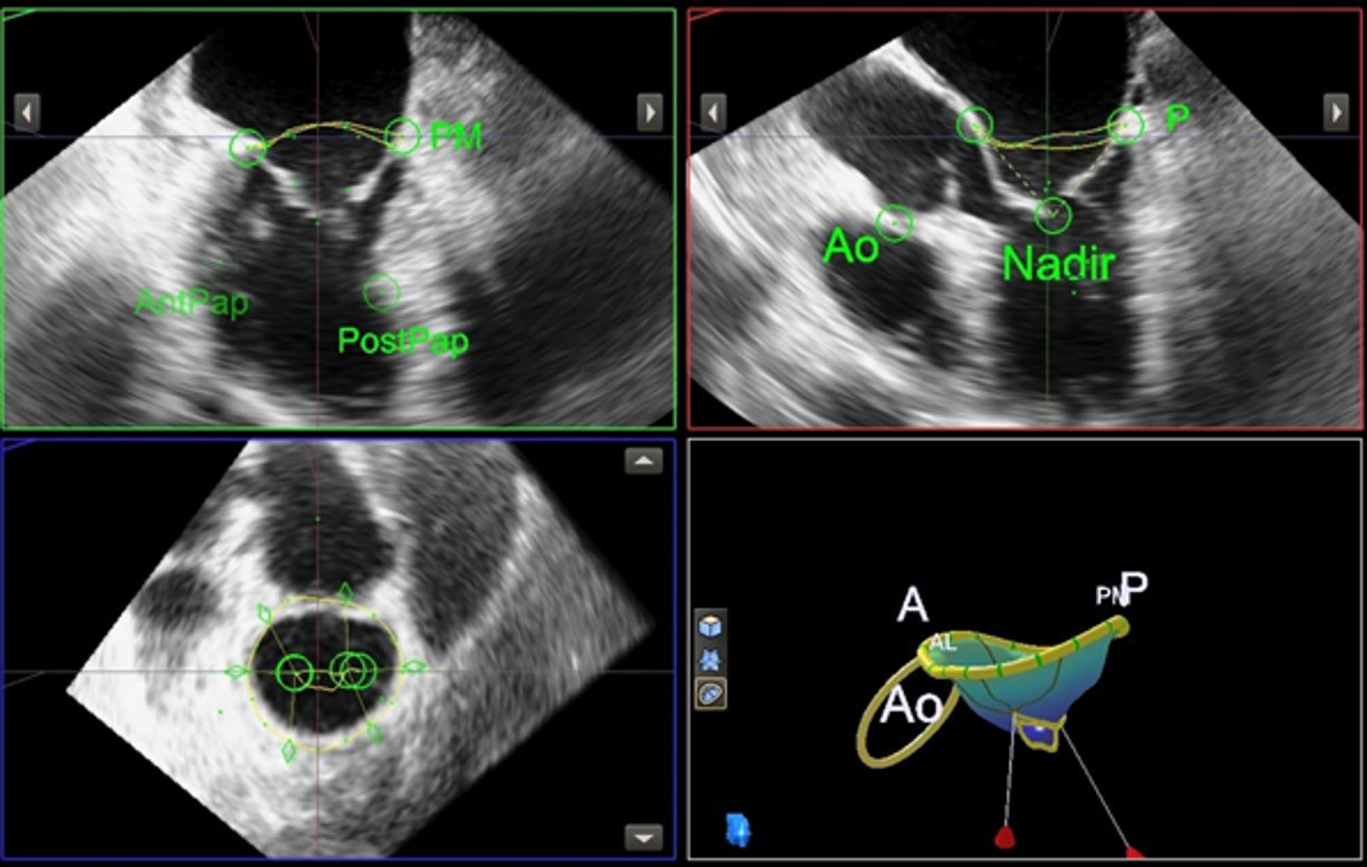
The Mitral Valve Navigation, a tool used to generate a three-dimensional model of the mitral valve

Acquired datasets were analysed offline using a commercially available semi-automated software programme (Mitral Valve Navigation, version 13, Philips Medical Systems). Analysis was performed by an experienced observer blinded to clinical data, following the methodology described by Mahmoud Elsayed et al. [8] (Fig. 1).

An early diastolic frame demonstrating maximum valve opening was selected as the reference. From this frame, three orthogonal planes were generated and adjusted to visualise the hinge points of the mitral leaflets. Four segmentation points were placed along the annulus: anterolateral, posteromedial, anterior, and posterior. Additionally, two points were placed at the nadir of the anterior leaflet and the distal aortic ring.

The MVN software used these landmarks to automatically reconstruct the mitral annulus and leaflets. A detailed manual review of the automatically generated annular contour was performed, with manual adjustments applied as needed. Commissural points were manually selected in the short-axis view, and leaflet edges were traced to delineate the valve orifice at maximum opening. Notably, the software labels this measurement as “MV MR orifice area” because the MVN software was originally designed to assess mitral valve prolapse regurgitant orifice during systole. However, Mahmoud Elsayed et al. proposed an adaptation of this tool by applying it to diastolic frame, enabling the evaluation of the mitral valve orifice during diastole, which corresponds to the stenotic orifice area.

**Fig. 2:**
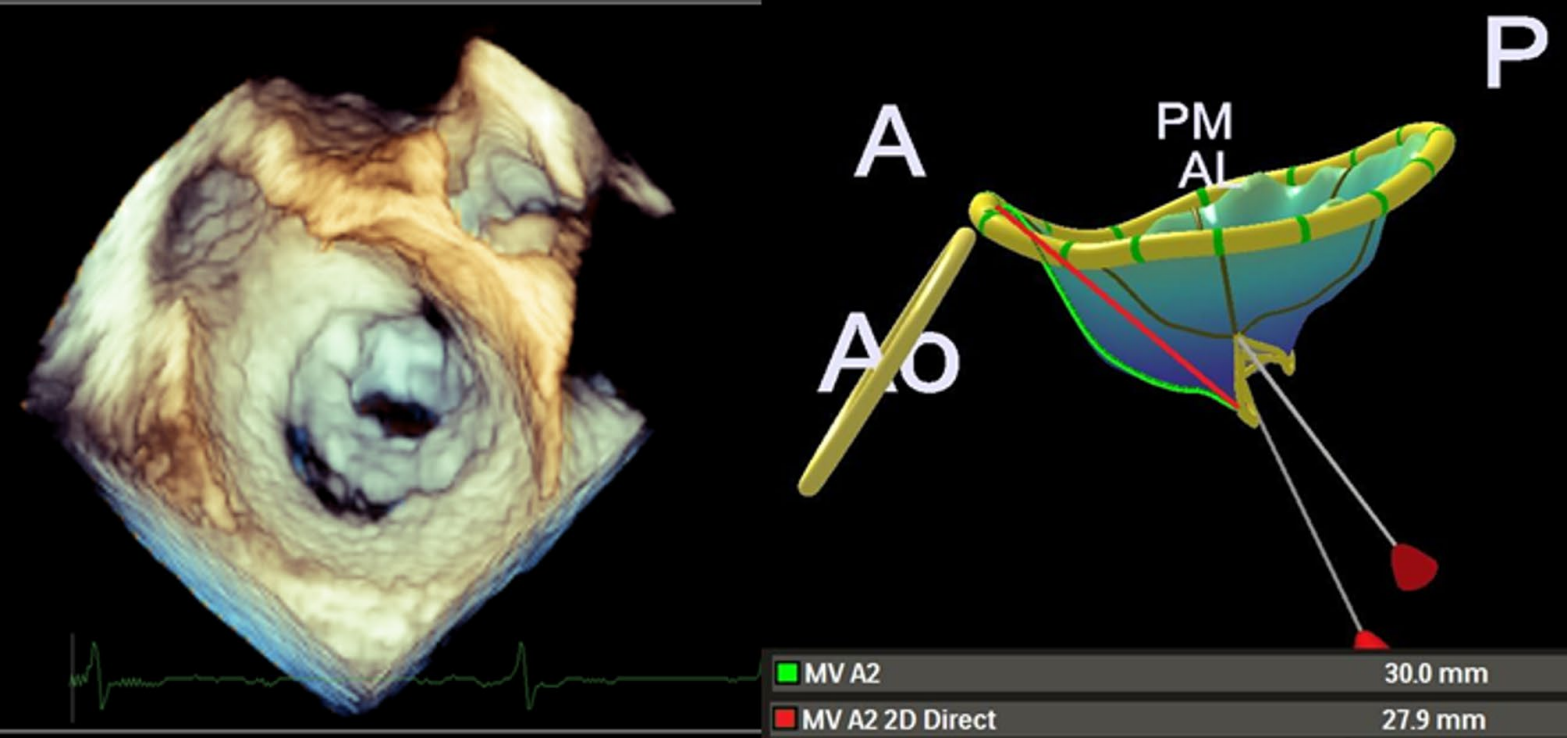
Three-dimensional transoesophageal echocardiography (3DTOE) in zoom mode demonstrating severe mitral stenosis with commissural fusion (left) and the corresponding reconstructed mitral valve model (right)

Following completion of the analysis, a three-dimensional model of the mitral valve was generated. The software produced a comprehensive quantitative report, including valve dimensions, angles, areas, and volumes. This 3D reconstruction provided in-depth information on mitral valve structure and function (Fig. 2).

### Assessment of mitral valve geometry

The reconstructed mitral valve was visualised as a color-coded 3D-rendered surface, producing a topographical map of the valve structure. The software automatically generated quantitative measurements for key 3D parameters, categorised as follows:

1. Annular geometry: The annular parameters were described by the anteroposterior diameter (the linear distance from the anterior to the posterior annulus), commissural width (the intercommissural distance between the posteromedial and anterolateral horns of the annulus), annular height (the maximum vertical distance between the highest and lowest points of the annular plane), circumference (the total perimeter of the annulus), and projected area (the surface area of the annulus as viewed from the atrial perspective).
2. Annular ellipticity: Annular ellipticity was calculated as the ratio of the anterolateral–posteromedial diameter to the anteroposterior diameter. This index reflects the geometric shape of the annulus and the degree of deviation from circularity.
3. Aortic–mitral non-planar angle: This angle was measured between the plane of the aortic orifice and the plane of the mitral valve annulus, providing insight into the spatial relationship between the two valve structures.
4. Anterior-posterior leaflet non-planar angle: This angle, defined between the anterior and posterior leaflets, represents the curvature (non-planarity) of the valve. A greater angle corresponds to a flatter leaflet configuration.

### Leaflet geometry

Leaflet morphology was assessed using multiple parameters, including tenting height and tenting volume. The lengths of the A2 and P2 scallops were measured directly as the linear distance from the midpoint of the annulus to the free edge of the leaflet in the fully opened position. The total leaflet area, as well as the individual areas of the anterior and posterior leaflets, were quantified. Leaflet asymmetry was estimated by calculating the ratio of posterior to anterior leaflet area.

**Fig. 3:**
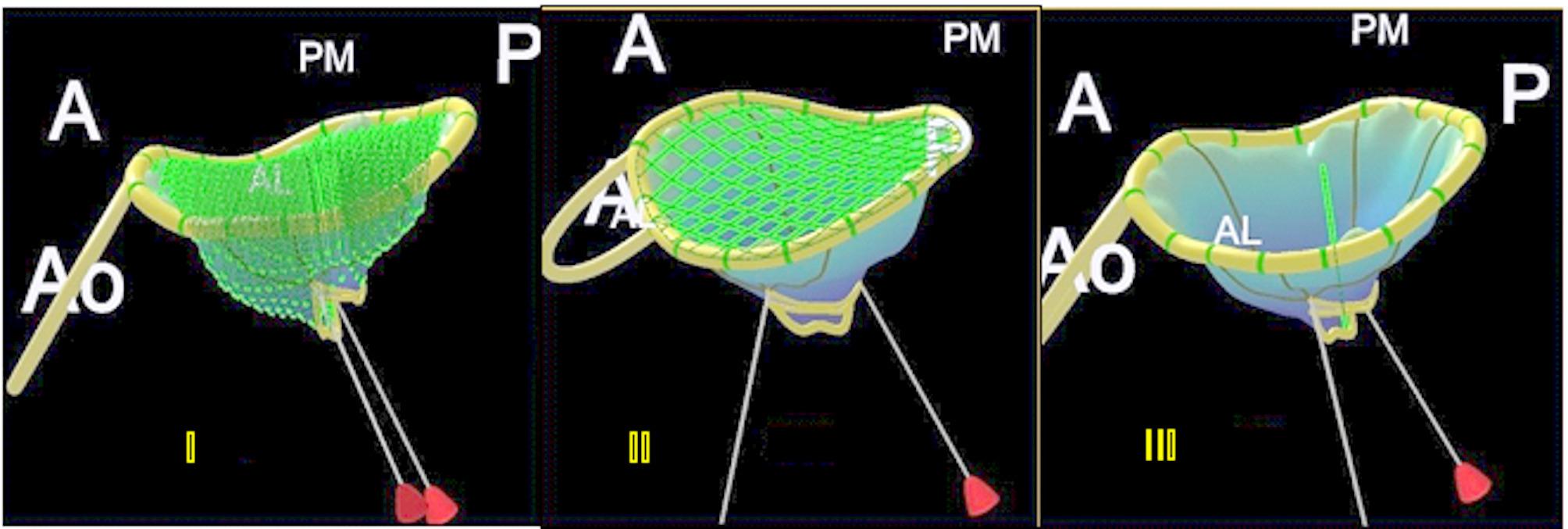
Calculation of the 3D Doming Index, defined as the ratio between the volume retained within the valvular structure and the theoretical volume of a cylinder based on annular area and tenting height (green line) I: Retained volume within the valve (tenting volume) II: Planar surface area of the mitral annulus (2D annular area) III: Vertical distance from the annular plane to the deepest point of the valve (tenting height)

In addition to the aforementioned geometric indices, the present study introduces a novel metric, the 3D Doming Index, designed to assess the haemodynamic severity of mitral stenosis. This index compares the volume retained within the valvular apparatus (tenting volume) to the theoretical volume of a cylinder generated using the two-dimensional annular area and tenting height (Fig. 3).

The equations used to define these geometric solids are provided in the Supplementary Material [14]. The final expression for the 3D Doming Index is as follows:

$$\:3D\:Doming\:index\:\left(ID\right)=2\frac{{V}_{valve}}{{A}_{annulus\:}\:\:{H}_{valve\:}\:\:}\:x\:\text{1,000}$$

Where *V valve* represents the volume retained within the valvular structure (tenting volume), *A annulus* is the area of the annulus, and *H valve* denotes the height from the annular plane to the point of maximum leaflet displacement (tenting height). The multiplication factor of 1,000 is applied to convert mm³ to mL, thereby standardising all volume-related parameters to the same unit of measurement.

**Fig. 4:**
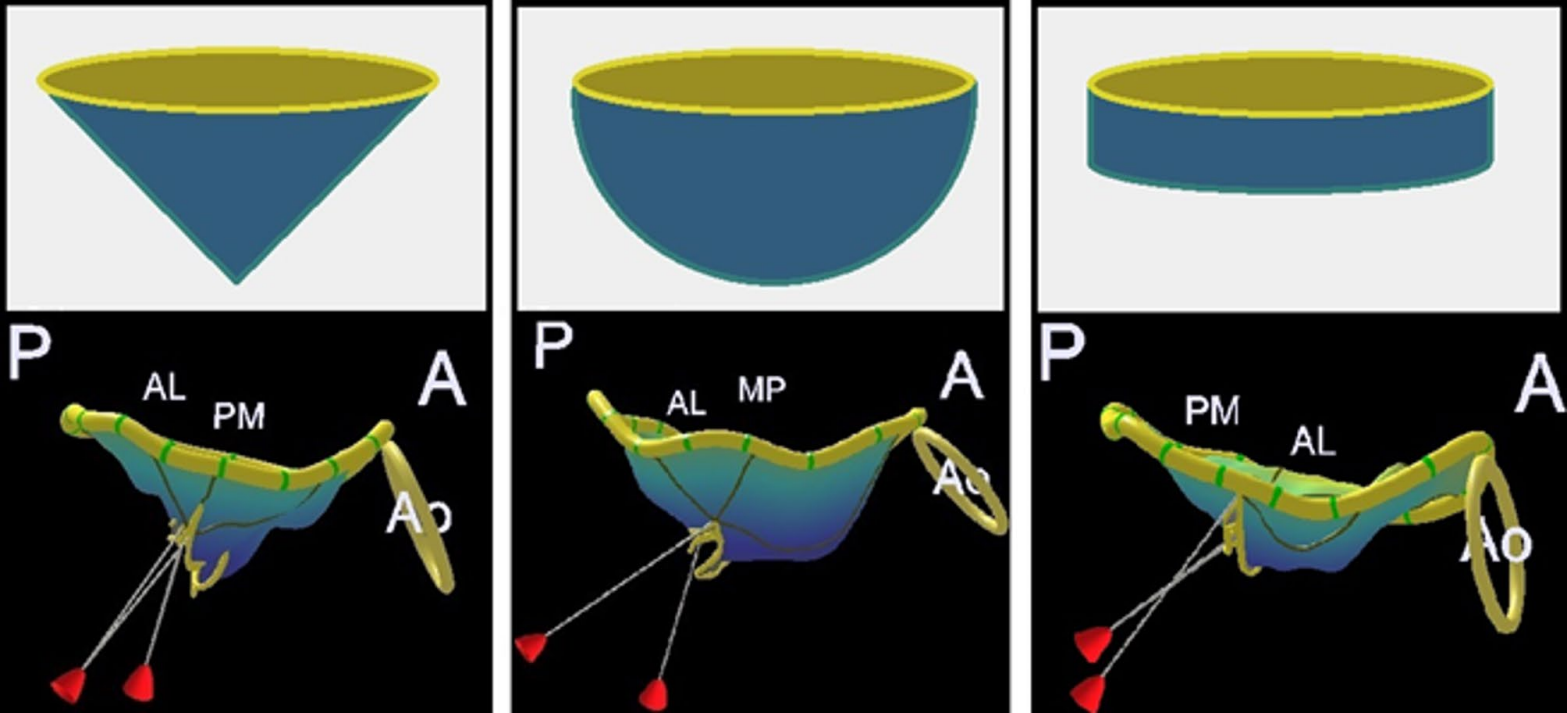
Geometric representations of the mitral valve **Top row**: Schematic illustrations of three distinct mitral valve geometries: doming (left), conical (centre), and planar (right) **Bottom row**: Corresponding 3D reconstructions derived from echocardiographic data. The first two valves (left and centre) have 3D Doming Index values of 0.25 and 0.38, respectively, despite having identical tenting heights (17 mm) and similar anterior–posterior leaflet non-planar angles (89° and 91°). In contrast, the planar valve (right) shows a lower 3D Doming Index of 0.18, a shorter tenting height (9 mm), and a greater anterior–posterior angle (104°)

The preliminary study by Gilon et al. [10] described three valve geometries, reconstructed to represent the morphological spectrum of mitral stenosis: planar, conical, and dome-shaped configurations (Fig. 4). For the purposes of the present study, the conical and dome-shaped models were selected for volumetric analysis, the former representing an intermediate anatomical form, and the latter corresponding to the structure with the greatest enclosed volume. The planar geometry was not used for volumetric calculations in our study, as it represents a configuration with minimal leaflet elevation and negligible retained volume. This shape lacks the 3D cavity necessary for accurately estimating tenting volume, which is central to the calculation of the 3D Doming Index.

**Fig. 5:**
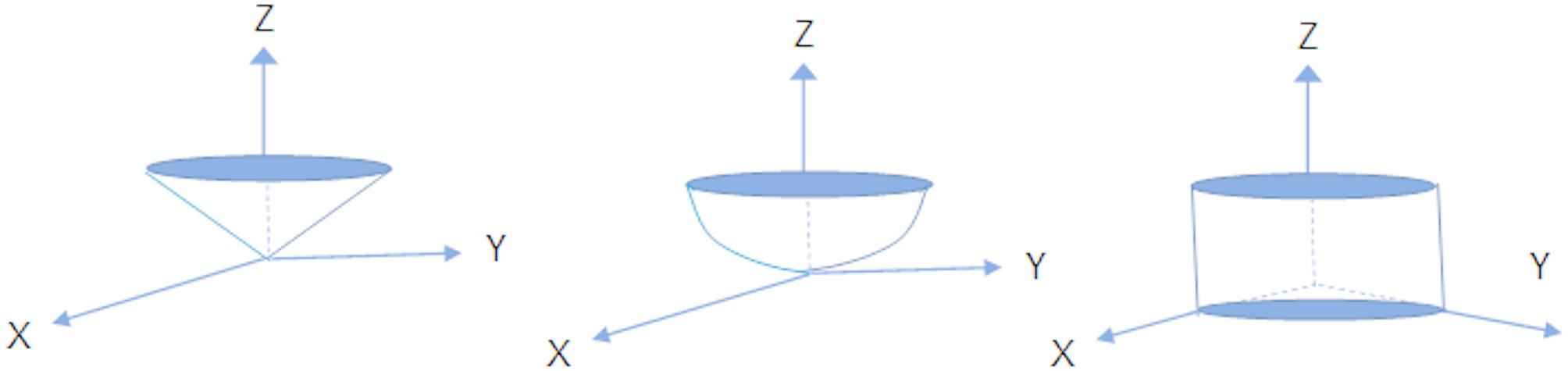
Geometric solids derived from the mitral valve annulus used for volumetric analysis

As illustrated in Fig. 4, valves with similar tenting heights and anterior–posterior leaflet non-planar angles may present markedly different 3D Doming Index values. In contrast, planar configurations are characterised by lower tenting heights and larger non-planar angles. A cylindrical model was employed as a reference for comparative purposes, as illustrated in Fig. 5.

### Statistical analysis

Baseline demographic characteristics and echocardiographic variables are presented as numbers and percentages for categorical data, and as mean ± standard deviation or median with interquartile range (IQR) for continuous data, as appropriate. The normality of continuous variables was assessed using the Shapiro-Wilk test. Correlations between continuous variables were explored using Pearson’s or Spearman’s correlation coefficients, depending on data distribution.

The mean transmitral pressure gradient was used as the principal haemodynamic variable to quantify the severity of valvular obstruction. Linear regression models were applied to identify factors associated with this gradient. Both univariable and multivariable linear regression analyses were performed. In the multivariable analysis, the dependent variable was the logarithm of the mean pressure gradient. Three sequential models were constructed.

The first model included determinants of mitral valve pressure gradient, including mitral valve area, heart rate, heart rhythm, left atrial size, C_n_, and the severity of concomitant mitral regurgitation. The second model incorporated all variables from Model 1, with additional adjustment for age and sex. The final model focused on geometric variables representing mitral valve shape, which were tested to determine their independent contribution to the variance in mean pressure gradient. Systolic pulmonary artery pressure was not included in the models, as higher mean transmitral gradients are generally associated with elevated pulmonary pressures. However, this relationship is non-linear and may be modulated by additional factors, such as pulmonary vascular resistance and C_n_ [15].

The explanatory power of each model was assessed using the coefficient of determination (R²). The incremental contribution of geometric variables to model performance was evaluated by comparing R² values across models. Potential interactions between variables were tested and included in the models where appropriate.

Model fit was assessed through residual analysis. Residual plots were examined for any systematic patterns, and the normality of residuals was confirmed using the Shapiro–Wilk test for both the overall and final models. Multicollinearity was checked by examining variance inflation factors; variables demonstrating clear interdependence were not included simultaneously in any model.

Reproducibility of the 3D Doming Index measurements was assessed using intraclass correlation coefficients (ICC) for intraobserver variability in a random subset of 20 patients. A two-tailed *p*-value of < 0.05 was considered statistically significant. All statistical analyses were conducted using the Statistical Package for the Social Sciences (SPSS), version 20.0 (IBM Corp., Armonk, NY, USA).

## Results

### Study population characteristics

A total of 186 patients were included, with a mean age of 47 years ± 12 years and 157 (84%) were female (Table 1). Most patients were symptomatic, presenting with varying degrees of dyspnoea, predominantly classified as New York Heart Association (NYHA) functional class II or III (76%). Atrial fibrillation was present in 72 patients (39%), and cerebrovascular events, including transient ischemic attack (TIA) and stroke, had occurred in 20%. At baseline, 43% of the patients were taking oral anticoagulants. Furthermore, a majority of the patients were on beta-blocker therapy (81%) and diuretic therapy (75%).

**Table 1:** Baseline characteristics of the study population

Variables		Values
Age, years		46.7 ± 12.4
Female		157 (84)
Weight, kg		68.8 ± 16.9
Height, m		1.60 ± 8.0
Body mass index, kg/m^2^		26.9 ± 6.0
Atrial fibrillation		72 (39)
Prior mitral valve intervention*		38 (21)
NYHA functional class	I/II	104 (56)
	III/IV	82 (44)
Right-sided heart failure		45 (24)
Ischaemic cerebrovascular events†		37 (20)
Medications, %	Diuretics	139 (75)
	β-Blockers	151 (81)
	ACE inhibitors	26 (14)
	Angiotensin receptor blockers	29 (16)
	Warfarin	79 (43)
	Penicillin benzathine	45 (24)
Blood pressure, mmHg	Systolic	117 ± 17
	Diastolic	76 ± 10
Heart rate, bpm		72 ± 14

Data are expressed as the mean value ± SD or number (percentage) of patients

* Surgical commissurotomy or percutaneous valvuloplasty

† Stroke or transient ischaemic attack at baseline

In the majority of the patients, mitral stenosis was associated with mild mitral regurgitation, whereas pure mitral stenosis was found in 26 patients (14%) with mean valve area of 1.1 ± 0.28 cm² (range, 0.36–2.18 cm²) as measured by planimetry. Mild aortic stenosis was detected in 53 patients (28%), with a mean pressure gradient of 12 mmHg. The echocardiographic characteristics of the study population are shown in Table 2. In the overall population, the mean transmitral gradient was 11.1 ± 4.5 mmHg, C_n_ was 4.8 ± 1.5 mL/mmHg, and systolic pulmonary artery pressure was 47.3 ± 19.5 mmHg. Moderate or severe tricuspid regurgitation was present in 30 patients (16%).

**Table 2:** Echocardiographic data of the study population

Variables			Values
Left atrial diameter, mm			50.4 ± 7.4
Left atrial volume index, mL/m²			62.5 ± 21.4
LV end-diastolic diameter (mm)			47.5 ± 6.9
LV end-systolic diameter (mm)			31.6 ± 5.0
LV ejection fraction (%)			58 ± 7
Mitral valve area by planimetry (cm^2^)			1.01 ± 0.28
Mitral stenosis severity by valve area	< 1cm^2^		93 (50)
	1-1.5 cm^2^		82 (44)
	> 1.5 cm^2^		11 (6)
Transmitral peak gradient, mmHg			19.5 ± 6.7
Transmitral mean gradient, mmHg			11.1 ± 4.5
Mitral valve morphology		Wilkins score	8 (5–11)
		Revisited echo score	2 (0–11)
Degree of mitral regurgitation		Absent	34 (18)
		Mild	148 (80)
		Moderate	4 (2)
RV function		RV basal diameter, mm	40.0 ± 6.5
		RV end-diastolic area, cm^2^	18.6 ± 5.7
		RV end-systolic area, cm^2^	10.3 ± 4.2
		RV fractional area change,%	45.1 ± 10.6
		RV peak systolic velocity, cm/s	10.1 ± 1.9
		RV myocardial performance index	0.40 ± 0.19
		Tricuspid annular motion, mm	18.0 ± 3.8
Right atrial area, cm^2^			18.5 ± 7.7
Systolic pulmonary artery pressure, mmHg			47.3 ± 19.5
Net atrioventricular compliance, mL/mmHg			4.8 ± 1.5
Moderate or severe tricuspid regurgitation			30 (16)

Data are expressed as the mean value ± SD or number (percentage) of patients

### Mitral valve geometry

The 3D-TOE-derived geometric variables are presented in Table 3.

**Table 3:** Three-dimensional mitral valve measurements in rheumatic mitral stenosis

Variables	Values	Range
**Annulus parameters**		
AL-PM diameter, mm	37 ± 4.7	24–50
AP diameter, mm	41 (38,44)	31–56
Annular ellipsicity, %	89 ± 9	63–111
Annulus 2D area*, mm^2^	1200 (1057,1370)	722–2206
Annulus 3D area, mm^2^	1246 (1097,1415)	737–2261
Circumferential 2D length*, mm	125 (117,133)	97–168
Circumferential 3D length**, mm	131 ± 13	100–174
Annulus height, mm	9 ± 2	3.0–14
Non-planar Ao-Mitral angle, °	129 ± 9	102–154
**Leaflet parameters**		
A2 length, mm	33 ± 5.1	21–49
A2 direct length, mm	30 ± 4.3	19–42
P2 length, mm	21 ± 3.7	10–30
P2 direct length, mm	19 ± 3.3	9–29
Anterior leaflet 3D area†, mm²	1042 (861,1182)	522–1790
Posterior leaflet 3D area†, mm²	683 (563,795)	328–1247
Total 3D leaflet area†	1747 (1471,1953)	963–3038
Non-planar A-P leaflet angle‡, °	88 ± 9	64–124
Tenting height‡, mm	15 ± 3.5	6–25
Tenting volume, mL	5.5 (4,7)	1.3–13
**Mitral Valve Orifice**		
Mitral valve area, cm²	0.90 (0.66,1.20)	0.26–2.36
Anterior valve edge length, mm	29 (25,35)	16–53
Posterior valve edge length, mm	27 (23,30)	14–51
Intercommissural diameter, mm	18 (16,20)	12–28
**Calculated geometrical parameters**		
3D Doming Index	0.28 ± 0.06	0.09–0.44
**Asymmetry**		
Leaflet area ratio	0.67 ± 0.14	0.29–1.13

Data are expressed as mean value ± SD or median (interquartile range), minimum- maximum values, as appropriate

* 2D circumferences and 2D areas are measurements in a projection plane

** 3D annulus area is the area of minimum surface spanning annulus

† 3D leaflet area means exposure area of leaflets

‡ Tenting height and non-planar A-P leaflet angle are parameters describing the “flat vs funnel” spectrum

A key parameter evaluated in this study was the 3D Doming Index, which had a mean value of 0.28 ± 0.06, with a range from 0.09 to 0.44. The anterior–posterior leaflet non-planar angle, representing the curvature between the anterior and posterior leaflets, had a mean value of 88 ± 9 degrees (range: 64–124 degrees).

The mean tenting height was 15 ± 3.5 mm. The volume-to-area ratio had a mean of 3.16 mm³/mm² (range: 0.79–5.06 mm³/mm²). There was a predominance of anterior leaflet area, with a leaflet area ratio (posterior/anterior) of 0.67, indicating that the anterior leaflet was generally larger. However, in some patients, the posterior leaflet area exceeded that of the anterior, with the ratio reaching a maximum of 1.13.

### Determinants of the mean transmitral pressure gradient

Variables associated with the mean transmitral pressure gradient are shown in Table 4. In a multivariable regression analysis, Model 1 included key determinants of pressure gradient across the mitral valve, namely, mitral valve area, heart rate, C_n_, and the severity of concomitant mitral regurgitation. In this model, mitral valve area was not independently associated with the mean pressure gradient. Model 2 incorporated age and female sex in addition to the variables in Model 1, which resulted in improved model performance.

**Table 4:** Factors associated with mean transmitral pressure gradient by multiple linear regression analysis

Variables	Beta-Coefficients	*p* Value	*R*^2^ *
**Model 1**			
Heart rate (bpm)	0.007	< 0.001	0.488
Atrial fibrillation	-0.237	< 0.001	
Mitral valve area (cm^2^)	-0.120	0.321	
Net atrioventricular compliance (C_n_)	-0.139	< 0.001	
Left atrial volume index (mL/m^2^)	0.005	< 0.001	
Mitral regurgitation severity	-0.144	0.030	
**Model 2** (adjusted for age and sex)			
Age (years)	-0.006	0.016	0.518
Female	-0.142	0.050	
Heart rate (bpm)	0.007	0.001	
Atrial fibrillation	-0.194	0.001	
Mitral valve area (cm^2^)	-0.044	0.713	
Net atrioventricular compliance (C_n_)	-0.145	< 0.001	
Left atrial volume index (mL/m^2^)	0.004	0.001	
Mitral regurgitation severity	-0.086	0.206	
**Model 3** (adjusted for 3D Doming index)			
Age (years)	-0.006	0.017	0.547
Female	-0.147	0.039	
Heart rate (bpm)	0.007	< 0.001	
Atrial fibrillation	-0.176	0.003	
Mitral valve area (cm^2^)	-0.055	0.642	
Net atrioventricular compliance (C_n_)	-0.142	< 0.001	
Left atrial volume index (mL/m^2^)	0.004	< 0.001	
Mitral regurgitation severity	-0.067	0.318	
3D Doming index	0.972	0.018	

The R^2^ values are cumulative, and the R^2^ value for the final model was 0.55

In the final model (Model 3), three-dimensional geometric variables were added. Among these, the 3D Doming Index was the only parameter independently associated with the mean transmitral pressure gradient, contributing to further improvement in model performance.

### Reproducibility

The variables contributing to the calculation of the 3D Doming Index (tenting volume, tenting height and 3D annular minimum area) were assessed for measurement variability. Excellent agreement was observed, with intraclass correlation coefficients of 0.92 for tenting height, 0.97 for tenting volume, 0.94 for 3D annular minimum area, and 0.86 for the 3D Doming Index (*p* < 0.001 for all analyses).

## Discussion

The current study investigates the influence of mitral valve morphology on haemodynamic parameters in rheumatic MS. The assessment of the 3D doming index provides valuable insights into the relationship between valvular geometry and the resulting haemodynamic burden of stenosis. These findings contribute to a better understanding of the pathophysiology of rheumatic MS and may have implications for clinical assessment and therapeutic decision-making.

The mitral valve is a complex, dynamic 3D structure that undergoes significant morphological changes in rheumatic MS. Commissural fusion, particularly involving the anterior leaflet, often results in a characteristic dome-shaped configuration [16]. This anatomical feature has previously been recognised as a predictor of successful percutaneous mitral valvuloplasty, underscoring its clinical relevance [13]. Despite this understanding, there is a lack of literature describing the direct correlation between mitral valve geometry and transvalvular pressure gradient in vivo.

A pioneering in vitro study by Gilon et al. addressed this knowledge gap by demonstrating that the 3D geometry of the proximal leaflet segment in relation to the stenotic orifice significantly affects the contraction coefficient and, consequently, the transmitral pressure gradient [10]. Their experimental models showed that dome-shaped valves had a higher contraction coefficient than flattened valves, which translated into an increased effective valve area. Conversely, flatter configurations were associated with higher pressure gradients and reduced valve opening, contributing to greater obstruction [5, 10].

One of the major challenges in evaluating mitral valve morphology in clinical practice lies in the geometric assumptions often required in conventional echocardiography. These include treating the left ventricular outflow tract as circular, the mitral annulus as a flat plane, and the flow convergence region as a symmetrical hemisphere. In reality, however, the mitral annulus possesses a saddle-shaped geometry, and the leaflet edges are often irregular, resulting in a non-planar and highly variable mitral valve orifice. These anatomical complexities may limit the accuracy of traditional haemodynamic assessment and highlight the potential utility of 3D imaging modalities in characterising valvular obstruction more precisely.

Recent advances in 3D echocardiographic techniques have transformed the evaluation of cardiac structures by allowing assessment based on true anatomical geometry. Mahmoud Elsayed et al. [8] conducted a study utilizing the Mitral Valve Navigation (MVN) tool, integrated within the Q-Lab software, to reconstruct 3D models of rheumatic mitral valves using diastolic frames. The MVN tool employed by Mahmoud Elsayed et al. accounted for the position of the commissures and accurately delineated the true mitral valve orifice, thereby providing a more anatomically precise representation of valve geometry. This approach demonstrated excellent correlation with invasively measured mitral valve area using Gorlin’s formula [8].

Beyond valve area quantification, the MVN method offers a comprehensive dataset, including tenting volume, tenting height, annular area, and total leaflet area, among other parameters. Utilising 3D modelling enables a more robust and reliable analysis, reducing the reliance on geometric assumptions inherent in conventional two-dimensional assessment [9]. The MVN method offers a significant advantage by incorporating the assessment of the commissures, thereby reducing the risk of underestimating the mitral valve area.

In contrast to conventional two-dimensional or three-dimensional planimetry, which does not account for the variation in depth between the distal leaflet tips and the proximal commissural points relative to the mitral annulus, the MVN approach considers these anatomical nuances, resulting in a more accurate three-dimensional representation of the valve. This enhanced precision may help explain why mitral valve area was not statistically significant in the multivariable models, which already included variables such as heart rate, presence of atrial fibrillation, and left atrial size and compliance (expressed as net atrioventricular compliance). These parameters, taken together, likely capture much of the haemodynamic burden traditionally attributed to valve area alone.

Nevertheless, despite technological advances in imaging, classifying mitral valve morphology according to the categories proposed by Gilon et al. [10] (planar, conical, and dome-shaped) based solely on 3D imaging remains a subjective endeavour. Due to the structural complexity of the mitral valve apparatus, such classification is prone to interobserver variability, with different operators potentially assigning different morphological types to the same valve. As a result, assessing the degree of obstruction based solely on perceived three-dimensional structure may introduce interpretative errors. This highlights the need for a more objective and reproducible parameter to evaluate valvular obstruction.

In this context, the 3D Doming Index was developed to provide a quantifiable and objective measure of valvular obstruction. It expresses the volume retained within the mitral valve apparatus (tenting volume) as a proportion of the maximum volume the structure could theoretically contain, defined by the annular area and tenting height. This dimensionless ratio reflects the degree to which the valve is domed or funnelled, with higher values indicating greater obstruction. By incorporating 3D geometry into a simplified formula, the 3D Doming Index offers a reproducible parameter that complements conventional markers and enhances the haemodynamic assessment of mitral stenosis.

### Clinical implications

Mitral valve area (MVA) is a classical parameter used to the severity of MS and guide treatment decisions [17]. However, the impact of a stenotic valve on intracardiac pressures is not solely determined by the cross-sectional area of the constricted orifice. It is also influenced by the three-dimensional geometry of the valvular structure.

Patients with similar MVA values may present with markedly different clinical profiles and transmitral pressure gradients, reflecting the limitations of relying on anatomic area alone [15]. This variability highlights the need for complementary parameters that more accurately reflect the functional severity of the stenosis.

The 3D doming index can be used to complement the assessment of MS severity. It not only incorporates valve area but also accounts for the full effect of the three-dimensional configuration of the mitral valve. By quantifying the volume retained within the valvular structure relative to its geometric capacity, the index provides a more comprehensive representation of the obstruction’s haemodynamic impact.

Clinically, the 3D Doming Index may be particularly useful in discordant cases, where symptoms or pressure gradients are not fully explained by valve area alone. While current guidelines recommend exercise stress echocardiography to clarify severity in such cases [17–19] the 3D Doming Index offers complementary anatomical information at rest, especially when stress imaging is not feasible or when valve geometry contributes significantly to obstruction. Its use may improve the overall precision of mitral stenosis assessment, and support more individualised patient management.

Nonetheless, its broader applicability and prognostic utility remain to be established. Prospective studies in larger, more diverse cohorts are required to validate the index’s reproducibility and to determine its ability to predict clinical outcomes. Such investigations will be essential to define how best to incorporate the 3D Doming Index into routine diagnostic and decision-making algorithms.

### Study limitations

Assessment of the mitral valve using a single frame limits the ability to capture the dynamic nature of leaflet and annular motion throughout the cardiac cycle. Nonetheless, selection of an early diastolic frame, corresponding to the point of maximal valve opening, provides a consistent and representative geometric configuration for evaluating stenotic morphology.

The observed correlation between the 3D Doming Index and mean transmitral pressure gradients may be particularly applicable to populations in endemic regions, where patients often present with pliable valves. In contrast, older individuals or those in non-endemic, developed settings, where fibrosis and calcification are more prevalent, may exhibit a different haemodynamic response, potentially limiting the generalisability of our findings [20].

Additionally, the geometric models used to calculate the 3D Doming Index were based on idealised mathematical solids (e.g. cone, dome, and cylinder). In practice, cardiac structures frequently deviate from these perfect geometries [14]. As such, the derived index should be interpreted as an approximation of the true anatomical volume relationships, acknowledging inherent variability in real-world valve morphology.

Additionally, this 3D-derived measurement is highly dependent on optimal image acquisition and requires dedicated post-processing software, which may not be readily available in low-resource settings, where rheumatic MS is most prevalent. This limitation may constrain the broader applicability of the 3D Doming Index in routine clinical practice, particularly in endemic, resource-constrained environments.

## Conclusions

In rheumatic mitral stenosis, the 3D Doming Index derived from transoesophageal 3D echocardiography demonstrates a significant correlation with mean transmitral pressure gradients. A higher index value is independently associated with greater haemodynamic burden, reflecting increased stenosis severity beyond traditional determinants of transvalvular flow. By translating complex geometric analysis into a simplified and reproducible metric, the 3D Doming Index offers a promising complementary parameter for assessing the functional severity of mitral valve stenosis.

## Supplementary Information

Below is the link to the electronic supplementary material.

Supplementary Material 1

Supplementary Material 2

## Data Availability

No datasets were generated or analysed during the current study.
