# Supplementary figures and images for: The value of a novel three-dimensional mitral valve index in the assessment of the haemodynamic severity of rheumatic mitral stenosis
Source: Echo Res Pract. 2025 Nov 12;12:33. doi: 10.1186/s44156-025-00094-z (PMC12606853; doi:10.1186/s44156-025-00094-z)

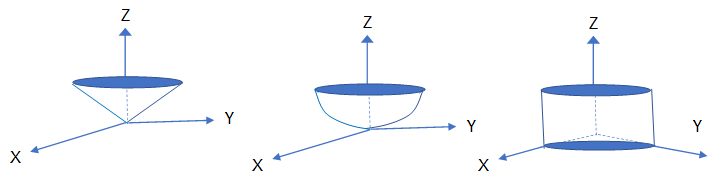

Supplement: Supplementary file 1 — Supplementary Material 1 [file 44156_2025_94_MOESM1_ESM.tif]
