# Supplementary material for: The value of a novel three-dimensional mitral valve index in the assessment of the haemodynamic severity of rheumatic mitral stenosis
Source: Echo Res Pract. 2025 Nov 12;12:33. doi: 10.1186/s44156-025-00094-z (PMC12606853; doi:10.1186/s44156-025-00094-z)
Supplement: Supplementary file 2 — Supplementary Material 2 [file 44156_2025_94_MOESM2_ESM.docx]

**Supplemental material**

The solids are represented by equations (1), (2), and (3), which are, respectively, the specific equations of a cone, a dome (paraboloid), and a cylinder. These solids will be formed from the opening of the mitral annulus. Thus, "R" represents the radius of the annulus, and "H" represents valvar height (tenting height).

$\frac{X^{2}}{R^{2}}+ \frac{y^{2}}{R^{2}}= \frac{z^{2}}{H^{2}}$ (1)

$\frac{X^{2}}{R^{2}}+ \frac{y^{2}}{R^{2}}= \frac{z}{H}$ (2)

$\frac{x^{2}}{R^{2}}+\frac{y^{2}}{R^{2}}= 1$ (3)

The volumes (V) of each solid can be calculated through triple integrals (volume integrals), where the integration limits of each are defined by the three equations presented earlier. Equations (4), (5), and (6) provide the mathematical model for these calculations[^21^](#_ENREF_10).

$V_{cone}=\int_{0}^{2\pi} \int_{0}^{R} \int_{\frac{H}{R} r}^{H} r dz dr d\theta$ (4)

$V_{dome}=\int_{0}^{2\pi} \int_{0}^{R} \int_{\frac{H}{R^{2}} r}^{H} r dz dr d\theta$ (5)

$V_{\mathrm{cylinder}}=\int_{0}^{2\pi} \int_{0}^{R} \int_{0}^{H} r dz dr d\theta$ (6)

The results of the integrals are presented in equations (7), (8), and (9).

$V_{cone}=\frac{{\pi R}^{2} H}{3}$ (7)

$V_{dome}=\frac{{\pi R}^{2} H}{2}$ (8)

$V_{\mathrm{cylinder}}={\pi R}^{2} H$ (9)

From the obtained results, it is possible to establish the proportion between the volumes (ID = 3D doming index), by respectively dividing the volume of the cone and the dome by a reference volume, which is that of the cylinder. Equations (10) and (11) present this ratio. Figure 1 provides a visual indication of the comparisons.

${ID}_{\mathrm{intermediate}}=\frac{V_{cone}}{V_{\mathrm{cylinder}}}=\frac{\frac{{\pi R}^{2} H}{3}}{{\pi R}^{2} H}= \frac{{\pi R}^{2} H}{3} \frac{1}{{\pi R}^{2} H}= \frac{{\pi R}^{2} H}{{\pi R}^{2} H} \frac{1}{3}= \frac{1}{3}$ (10)

${ID}_{\mathrm{major}}=\frac{V_{dome}}{V_{\mathrm{cylinder}}}=\frac{\frac{{\pi R}^{2} H}{2}}{{\pi R}^{2} H}= \frac{{\pi R}^{2} H}{2} \frac{1}{{\pi R}^{2} H}= \frac{{\pi R}^{2} H}{{\pi R}^{2} H} \frac{1}{2}= \frac{1}{2}$ (11)

It can be observed that the maximum value of the 3D doming index is ID = 0.5. To normalize this measure to values between zero and one (from 0 to 100%), normalization should be performed. This involves dividing the intermediate index by the maximum value found. Thus, the following result is obtained:

$ID=\frac{{ID}_{\mathrm{intermediate}}}{{ID}_{\mathrm{major}}}=\frac{\frac{V_{cone}}{V_{\mathrm{cylinder}}}}{\frac{V_{dome}}{V_{\mathrm{cylinder}}}}= \frac{V_{cone}}{V_{\mathrm{cylinder}}} x \frac{V_{\mathrm{cylinder}}}{V_{dome}} =\frac{V_{\mathrm{cylinder}}}{V_{\mathrm{cylinder}}} x \frac{V_{cone}}{V_{dome}}=\frac{V_{cone}}{V_{dome}}$ (12)

By normalizing the equation, it can be observed that the volume of the cylinder is replaced by the volume of the dome. This is what gives the index its name.

Now, to generalize Equation (12), the volume of the cone (intermediate value) is substituted by the volume of the valvular apparatus (tenting volume).

$ID=\frac{V_{valve}}{V_{dome}}$ (13)

Substituting Equation (8) into Equation (13) yields:

$ID=\frac{V_{valve}}{\frac{{\pi R}^{2} H}{2}}=2\frac{V_{valve}}{{\pi R}^{2} H}$ (14)

The denominator of Equation (14) is exactly the volume of a cylinder, as can be observed in Equation (9). Therefore:

$ID=2\frac{V_{valve}}{V_{\mathrm{cylinder}}}$ (15)

The volume of the cylinder can be defined as the multiplication of the area (A) of the mitral annulus by the height (H) of the valve.

$ID=2\frac{V_{valve}}{A_{\mathrm{annulus}} H_{valve}}$ (16)

Equation (16) does not alter the result of Equation (13), but presents it with parameters that are already known. However, the multiplication of the denominator results in a volume whose unit of measurement is given in "mm3," which is different from the unit of measurement of the valvular volume (ml). Therefore, an adjustment factor is necessary, as presented below.

$ID=2\frac{V_{valve}}{A_{annulus} H_{valve}} x 1.000$ (17)

Thus, Equation (17) represents the normalized value of ID.


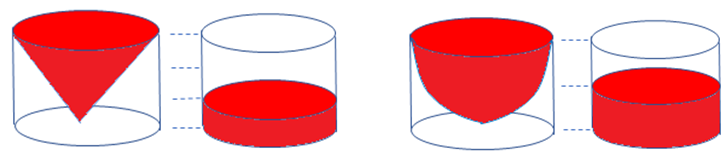


**Figure 1:** The volume of a cone, when inserted into a cylinder of the same diameter, will occupy one-third of the volume of the cylinder. Conversely, this same relationship for a dome structure is 50% of the occupied volume.

**Reference:**

Stawrt, J., Clegg, D., & Watson, S. 2020. Calculus. Book. Cengage Learning; 9th edition (April 30, 2020).
